# Proteomics of novel induced pluripotent stem cell-derived vascular endothelial cells reveal extensive similarity with an immortalized human endothelial cell line

**DOI:** 10.1152/physiolgenomics.00166.2022

**Published:** 2023-06-12

**Authors:** Nethika R. Ariyasinghe, Roberta de Souza Santos, Andrew Gross, Arwin Aghamaleky-Sarvestany, Simion Kreimer, Sean Escopete, Sarah J. Parker, Dhruv Sareen

**Affiliations:** ^1^Board of Governors Regenerative Medicine Institute, Cedars-Sinai Medical Center, Los Angeles, California, United States; ^2^Cedars-Sinai Biomanufacturing Center, Cedars-Sinai Medical Center, Los Angeles, California, United States; ^3^Department of Cardiology, Smidt Heart Institute, Cedars-Sinai Medical Center, Los Angeles, California, United States; ^4^iPSC Core, Cedars-Sinai Medical Center, Los Angeles, California, United States; ^5^Board of Governors Innovation Center, Cedars-Sinai Medical Center, Los Angeles, California, United States; ^6^Department of Biomedical Sciences, Cedars-Sinai Medical Center, Los Angeles, California, United States

**Keywords:** differentiation of stem cells, proteomics, stem cells, vascular endothelial cells, vascular tissue regeneration

## Abstract

The vascular endothelium constitutes the inner lining of the blood vessel, and malfunction and injuries of the endothelium can cause cardiovascular diseases as well as other diseases including stroke, tumor growth, and chronic kidney failure. Generation of effective sources to replace injured endothelial cells (ECs) could have significant clinical impact, and somatic cell sources like peripheral or cord blood cannot credibly supply enough endothelial cell progenitors for multitude of treatments. Pluripotent stem cells are a promising source for a reliable EC supply, which have the potential to restore tissue function and treat vascular diseases. We have developed methods to differentiate induced pluripotent stem cells (iPSCs) efficiently and robustly across multiple iPSC lines into nontissue-specific pan vascular ECs (iECs) with high purity. These iECs present with canonical endothelial cell markers and exhibit measures of endothelial cell functionality with the uptake of Dil fluorescent dye-labeled acetylated low-density lipoprotein (Dil-Ac-LDL) and tube formation. Using proteomic analysis, we revealed that the iECs are more proteomically similar to established human umbilical vein ECs (HUVECs) than to iPSCs. Posttranslational modifications (PTMs) were most shared between HUVECs and iECs, and potential targets for increasing the proteomic similarity of iECs to HUVECs were identified. Here we demonstrate an efficient robust method to differentiate iPSCs into functional ECs, and for the first time provide a comprehensive protein expression profile of iECs, which indicates their similarities with a widely used immortalized HUVECs, allowing for further mechanistic studies of EC development, signaling, and metabolism for future regenerative applications.

**NEW & NOTEWORTHY** We have developed methods to differentiate induced pluripotent stem cells (iPSCs) across multiple iPSC lines into nontissue-specific pan vascular ECs (iECs) and demonstrated the proteomic similarity of these cells to a widely used endothelial cell line (HUVECs). We also identified posttranslational modifications and targets for increasing the proteomic similarity of iECs to HUVECs. In the future, iECs can be used to study EC development, signaling, and metabolism for future regenerative applications.

## INTRODUCTION

The vascular endothelium is a monolayer of endothelial cells (ECs) that constitutes the inner lining of blood vessels, such as arteries, veins, and capillaries. It is considered as an active organ, and the main functions of the endothelium are related to controlling blood flow, vascular tone, platelet aggregation, as well as regulation of immunity, inflammation, and angiogenesis (1). The endothelium serves as the delivery system between organs and regulates access of molecules to tissues including oxygen, nutrients, growth factors, and hormones, thus forming a vital component toward restoration and regeneration of any injured tissue (2). Injuries in the endothelium and/or dysfunctions linked to improper synthesis or release of endothelial factors that contribute to blood fluidity are key factors to the initiation or propagation of existing cardiovascular pathogenesis (1). Importantly, cardiovascular diseases are the leading cause of death globally, taking 17.9 million lives each year, which represents 32% of all global deaths (3). Other diseases are also associated with the malfunction or injuries in the endothelium such as sepsis, stroke, tumor growth, metastasis, insulin resistance, venous thrombosis, chronic kidney failure, and others (4). Thus, the generation of effective sources to replace impaired endothelial cells (ECs) in vascular diseases seems urgent to restore angiogenesis or implement vasculogenesis in damaged tissues.

Generating and expanding new cells from adult differentiated tissues has proven to be a major limitation. Somatic human cells like ECs are not designed to expand in culture and rapidly lose the ability to replicate in sufficient numbers to scale up. Isolating ECs from somatic cell sources like peripheral or cord blood is likely to provide insufficient cell numbers for regenerative therapy treatments, as the effective treatment of ischemia in an average adult patient would require ∼12 L of blood to generate sufficient endothelial progenitor cells (EPCs) (5). A promising source for EC generation is through the differentiation of stem cells. Human pluripotent stem cells (PSCs) can self-renew indefinitely and differentiate into all cell types, providing tremendous promise for use in regenerative medicine. The utility of pluripotent human embryonic stem cells (ESCs) is limited by ethical issues associated with destroying a human embryo and the likelihood of allograft rejection by the recipient’s immune system. Induced pluripotent stem cells (iPSCs) have similar qualities as ESCs (6, 7), but avoid ethical issues as they are obtained through the reprogramming of adult cells. iPSCs can also be used to generate haplotype-matched banks or even for autologous cell transplantation, in which the cells are removed from a donor, manipulated, stored, and later given back to that same individual. With the advancement of iPSC technology (8), it is now possible to generate limitless iPSC-derived endothelial cells (iECs) for cell transplantation or tissue-engineering strategies (9, 10). Therefore, iPSC-derived endothelial cells can provide a readily available off-the-shelf solution. Upon engraftment, it is expected that ESCs would induce significant revascularization and contribute significantly to the repair of the damage to tissues (11). After integrating with the host vasculature networks, iEC therapy would replace impaired vascularity in damaged tissue and restore blood flow with critical nutrient transport, hemostasis, and immune processes, thus rescuing tissue functionality (12, 13).

To realize this clinical potential, existing methods for differentiating iPSCs into EC phenotypes reliably across multiple iPSC lines require improved efficiency to enhance purity and quality of the ECs generated. Diverse approaches have been published to differentiate pluripotent stem cells into endothelial cells (ECs), including coculture systems (14), three-dimensional (3-D) embryoid bodies (15), direct differentiation using growth factors and small molecules (16–18), and genetic manipulation (19, 20), which give rise to EC populations with different levels of purity (21). To support Investigational New Drug (IND)-enabling clinical trials for regenerative applications, a good manufacturing practice (cGMP)-compliant method that generates differentiated ECs from iPSCs reproducibly and reliably across multiple iPSC lines is critical. To date, validation of differentiated cell phenotypes has relied exclusively on transcript and/or antibody-based measurements of selected protein expression of selected markers, sometimes coupled with a larger scale transcriptomic analysis. Although mRNA levels can certainly be informative to cell states, there are currently no studies that have examined how the proteome shifts as iPSC are differentiated toward EC phenotypes, and whether iECs approach a proteomic state similar to a primary EC cell type. Furthermore, focus on RNA precludes study of important posttranslational modifications (PTMs) that may participate in key regulatory processes during cell differentiation or pluripotency maintenance. Here we describe a method to generate functional iECs with high efficiency (>80% CD144^+^/CD31^+^ cells) in 3 wk consistently across multiple donor iPSC lines without requiring cell sorting. In addition to reporting canonical indicators of iEC phenotype, we generated a comprehensive proteomic characterization of the iECs and observed extensive similarities between iECs and a widely used immortalized primary EC cell type, human umbilical vein endothelial cells (HUVECs). Notably, proteomics provides valuable information about the expression, function, identification, interaction, and structure of proteins (22), and to our knowledge, this is the first time that a study has demonstrated such an extensive proteomic analysis, including examination of key posttranslational modifications (PTMs), of ECs generated from iPSCs.

## METHODS

### iPSC Generation

The iPSC lines used in this study were generated from healthy male and female controls by the iPSC Core at Cedars-Sinai Biomanufacturing Center. These control iPSC lines were generated from the peripheral blood mononuclear cells (PBMCs) using nonintegrating oriP/EBNA1-based episomal plasmid vectors as previously described (23). This approach resulted in <5% of abnormal karyotypes of iPSCs. All undifferentiated iPSCs were maintained in mTeSR^+^ medium (STEMCELL Technologies) onto BD Matrigel matrix-coated plates. The cell lines used in this study are summarized at Supplemental Table S1. The Cedars-Sinai Biomanufacturing Center tests monthly for mycoplasma contamination on all cell lines and all lines in this study tested free of mycoplasma contamination. The reprogramming of iPSCs and differentiation protocols were carried out in accordance with the guidelines approved by Stem Cell Research Oversight (SCRO) committee and Institutional Review Board (IRB), under the auspices of IRB-SCRO Protocols Pro00036896 (Sareen Stem Cell Program) and Pro00032834 (iPSC Core Repository and Stem Cell Program).

### Differentiation of Vascular Endothelial Cells from iPSCs

For the generation of vascular endothelial cells (ECs) from iPSCs (iECs), we have adapted a previous protocol (24) to make our “in-house” robust and efficient protocol. In brief, iPSCs (OCT4 expression> 90%) were plated in planar onto Matrigel-coated plates as small colonies of cells, and 3 days later they were induced into mesoderm (ME *phase I*) using CHIR99021(6 μM, Xcess Bio) for 2 days. Next, vascular progenitors (VP *phase II*) were generated using a combination of bone morphogenic protein 4 (BMP4; 25 ng/mL, R&D Systems), fibroblast growth factor 2 (FGF2; 10 ng/mL, PeproTech), and vascular endothelial growth factor (VEGF_165_) (50 ng/mL, PeproTech) for another 2 days. After that, when the majority of the VPs presented a cobblestone-like morphology in the periphery of the original iPSC colonies, cells were dissociated with Accutase (Sigma), and the cells in the periphery that easily lifted were replated in planar onto Matrigel-coated plates at a density of 10,000 cells/cm^2^ with VEGF_165_ (50 ng/mL) and Y-27632 (10 μM, STEMCELL Tech) to induce EC progenitors (ECP *phase III*) for 7 days, changing media every other day. For purification and maturation of iECs (*phase IV*), iECs were dissociated at *day 11* and replated at the same cell density onto Matrigel-coated plates with VEGF_165_ (50 ng/mL) and media were changed every other day for 10 days. This process was repeated on *day 21* and extended for another 10 days if necessary. The base medium used for *phases I* and *II* was STEMdiffAPEL2 (STEMCELL Tech) and for *phases III* and *IV*, EC growth medium MV2 (ECGM-MV2; PromoCell) was used. For imaging purposes, cells were replated onto Matrigel-coated 96-well plates at a density of 100,000 cells/cm^2^ at *day 4* for imaging on *day 11* or plated at *day 11* for imaging on *day 21*. Human umbilical vein endothelial cells (HUVECs) were used as a positive control in some experiments and were fed with iEC *phase IV* complete media as described earlier.

### Tube Formation In Vitro Assay

For the tube formation assay, iECs on *day 21* were dissociated and replated onto a solid layer of growth factor-reduced Matrigel (Corning) in 96-well plates (15,000 cells/well) using *phase IV* complete media plus Y-27632. Bright-field images were taken 17 h after seeding (24).

### Dil-Acetylated LDL Uptake Assay

Dil fluorescent dye-labeled acetylated low-density lipoprotein (Dil-ac-LDL; 10 μg/mL, Cell Applications) was added to *phase IV* medium of iECs at *day 11* or *day 21* and incubated for 4 h at 37°C, as described previously (24). Cells were then washed with PBS, fixed, and stained with DAPI. Images were taken with ImageXpress Micro XLS (Molecular Devices) and analyzed using ImageJ software. HUVECs fed with *phase IV* medium were used as positive control, and iPSCs fed with mTeSR^+^ medium were used as negative control.

### Immunofluorescence

Cells were first fixed with 4% paraformaldehyde (PFA) in phosphate-buffered saline (PBS) for 20 min and subsequently washed two times with PBS. Fixed cells were then permeabilized and blocked for 1 h in a “blocking buffer” containing PBS with 10% donkey serum (Millipore) and 0.1% Triton-X (Bio-Rad). Primary antibodies were diluted in the blocking buffer and kept on cells overnight at 4°C. The following primary antibodies and dilutions were used: VEGF-A (1:100, Abcam), CD31 (1:100, Cell Signaling), CD144 (1:100, Abcam), and VGFR2 (1:100, Cell Signaling). The next day, after thorough washing using PBS with 0.1% Tween-20 (Thermo Fisher), cells were incubated with appropriate species-specific Alexa Fluor-conjugated secondary antibodies (Thermo Fisher) diluted in the blocking buffer (1:1,000) for 1 h at room temperature. After washing in PBS with 0.1% Tween-20, cells were incubated with DAPI diluted in PBS (1:2,500) for 15 min. Immunofluorescence images were taken using appropriate fluorescent filters using ImageXpress Micro XLS and analyzed using ImageJ software.

### Real-Time qPCR Analysis

Relative gene expression was quantified using real-time qPCR. For this, cells were washed with PBS and the total RNA was extracted and isolated using Quick-RNA MiniPrep Kit (Zymo Research), according to the manufacturer’s instructions. The concentration and purity of RNA were determined by spectrophotometric analysis (NanoDrop, Thermo Fisher), and all samples had a A_260/280_ ratio around 2.0 (25). Afterward, RNA (1 μg) was treated with DNAse (Thermo Fisher) and then reverse transcribed to cDNA with oligo(dT) using the High-Capacity cDNA Reverse Transcription Kit (Thermo Fisher). Real-time qPCR was performed in three replicates using SYBR Green master mix (Applied Biosystems) and primer sequences specific to each gene (Supplemental Table S2) and run on a CFX384 Real-Time System (Bio-Rad). Human *RPL13* was used as a reference gene and relative expression was determined using 2^−ΔΔCt^ method.

### Flow Cytometry Analysis

Cells were singularized using Accutase and filtered using a 70-μm nylon mesh. Cells were washed once with PBS plus 10% FBS (washing buffer), spun down at 300 *g* for 5 min (4°C) and resuspended in the washing buffer for 20 min (blocking phase) on ice. After this, the following treatments were added for 30 min on ice: no antibodies for “unstained samples”; FITC mouse anti-human CD31 and Alexa Fluor 647 mouse anti-human CD144 for “double-stained samples”; and FITC mouse IgG1 κ isotype control (ISO) and Alexa Fluor 647 mouse-IgG1 κ isotype control for “double ISO samples.” After this, cells were washed three times in the washing buffer (300 *g* for 5 min for each wash, 4°C). Cells were then fixed with FBS + 4% PFA for 15 min on ice. To finalize, cells were again washed three times in the washing buffer and analyzed using Fluorescence Activated Cell Sorter (FACS) Attune NxT (Thermo Fisher). Double ISO samples were used for gating.

### MACS Sorting Analysis

Cells were singularized with Accutase, filtered using a 70-μm nylon mesh, and sorted on a magnetic-associated cell sorting (MACS) machine (Miltenyi Biotec) according to the manufacturer’s instructions. In brief, cells were centrifuged at 300 *g* for 3 min and then resuspended with 60 μL iEC *phase IV* complete media (as described earlier) plus 20 μL CD31 MicroBeads per 5 × 10^6^ cells for 15 min on ice. After this, 1 mL of medium was added and cells were centrifuged again. Cells were resuspended in 1 mL of MACS sorting buffer and proceeded to magnetic separation using “Possel” for positive selection, following instructions of the manufacturer (autoMACS Pro, Miltenyi Biotec). After this, “CD31 positive fraction,” “CD31 negative fraction,” and “unsorted cells” were proceeded with CD144 staining flow cytometry analysis as described earlier.

### Proteomic Sample Preparation and Mass Spectrometry Acquisition

Cell pellets were lysed using 8 M urea/5% SDS with 100 mM DTT. Protein concentration was determined by bicinchoninic acid (BCA) assay and 50 μg of protein per sample was aliquoted for further processing. Protein aliquots were processed, digested, and cleaned using the S-Trap system (Protifi). Peptides were dried following elution from the S-Trap columns, and dried peptides were resuspended at a concentration of 1 µg/µL for injection onto mass spectrometry (MS) system. One microgram of the generated peptides was analyzed by LC-MS. The peptides were loaded on a Phenomenex Polar C18 trapping column (75 um × 2 cm) and analyzed by a two hour analytical gradient on 200 cm Pharmafluidics uPAC column. The analytical gradient with 0.1% formic acid in water as mobile phase A and 0.1% formic acid in acetonitrile as mobile phase B was delivered as follows: starting with 4% B at 1.2 µL/min flowrate, held for 5 min, ramped to 8% B in 0.2 min, B was ramped to 30% until 90 min as the flowrate was decreased to 1.0 µL/min, B was ramped to 50% over 30 min at the 1.0 µL/min flowrate. After each run the system was flushed with 98% B and equilibrated back to aqueous using a 20 min cleaning method. MS data were acquired using data independent acquisition (DIA) in each data acquisition cycle a MS1 (precursor scan) was acquired at 120K resolution over the 400-1,000 m/z mass range, followed by 20 MS2 scans at 15K resolution in which 15 m/z isolation windows were sequentially isolated and fragmented to cover the 400-1,000 m/z range.

### Proteomic Analysis

Raw MS files were analyzed using the DIA-Neural Network (NN) platform (PMC 6949130), with files searched using a “library-free” strategy via two approaches: total protein analysis was done by searching files against an in silico-digested protein FASTA sequence database (UniProt reviewed and canonical human sequences) and for PTM analysis, a library of detectable peptides was generated using the FragPipe workflow, wherein the same raw files used for total protein analysis were used to generate pseudospectra that are then searched for presence of prespecified PTMs. In this study, we interrogated phosphorylation of threonine, serine, and tyrosine (T, S, and Y); acetylation of lysines (K) and *N*-termini; and mono- and dimethylation of arginines (R) and lysines. High-confident (FDR < 1%) pseudospectra identifications were consolidated into a library that was then used to perform a second round of DIA-Umpire quantitative extraction (26), and the quantified peak areas for each protein with identification *q* value <1% FDR were analyzed for differential abundance between cell types using Fragger (27). For PTM analyses, an additional filter was applied to include only peptides with >0.8 MS1 correlation score, meaning that the MS1 chromatogram correlated in coelution and shape with the MS2 fragments, thus minimizing chances that a called PTM peptide could be in reality the product of the unmodified form erroneously identified by any overlapping fragment patterns (not all of which may carry the mass shift for a given PTM). PTM validation was completed using percolator (28). PTM abundances from the filtered DIA-NN peptide tables were normalized to the total protein quantification for the gene product that a given peptide was assigned to. Quantitative comparison of PTMs was done using Philosopher (29), IonQuant (30), and simple *t* tests in Excel (Microsoft). Total proteome analyses were performed in R software. Principal component analysis (PCA) was performed using the prcomp package using default settings. Differential expression analysis was performed using the DESeq2 Bioconductor package, which was used to generate the data for volcano plots, which were plotted using Ggplot2. Differential expression analysis was conducted on proteins with complete observations across all samples in the comparison (*n* = 4,028 proteins between iEC and HUVECs vs. iPSC; *n* = 4,632 proteins between iEC and HUVEC comparison)

The Venn diagram was prepared by compiling a list of all proteins detected in more than six of the nine iEC samples, two of the three HUVEC samples, and five of the seven iPSC samples, and then running set comparisons to determine which proteins were shared or absent across the three groups. A heatmap was prepared by plotting the expression of all proteins differentially expressed between iEC and HUVEC samples versus iPSC samples with an adjusted *P* value of 0.05.

### Statistical Analyses

Data are presented as means ± standard error (SE). Statistical significance between groups was determined by one-way ANOVA followed by Dunnett’s posttest. Two-tailed paired Student’s test was used as appropriate. *P* values <0.05 were considered statistically significant. Statistical analyses and graphs were generated using GraphPad Prism 7 for Windows Software (GraphPad software).

## RESULTS

### Robust and Efficient Differentiation of iPSC-Derived Vascular Endothelial Cells across Five Different iPSC Lines

**Figure 1.**
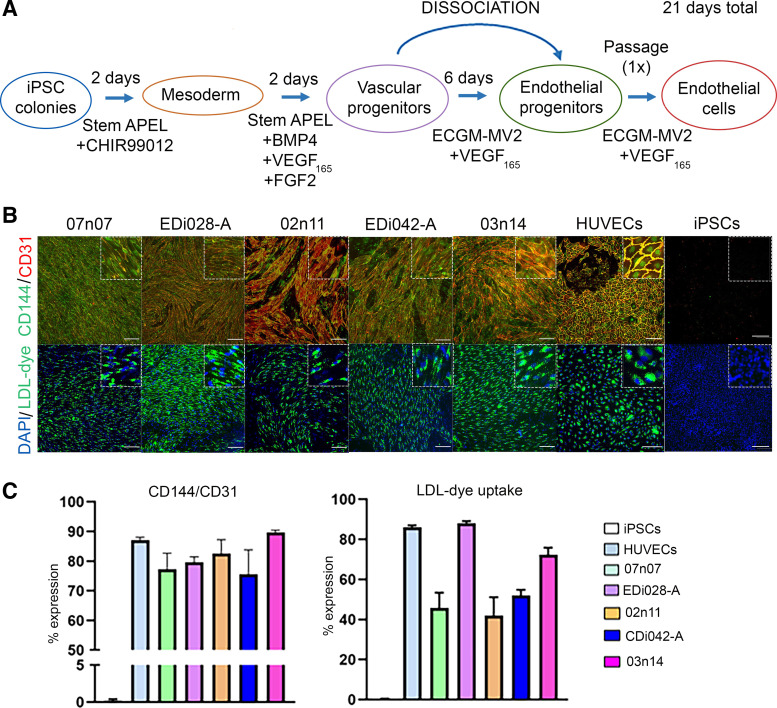
Efficient and reproducible generation of vascular endothelial cells (iECs) from 5 different iPSC lines. *A*: schematic of the iEC differentiation protocol. *B*: protein expression of vascular endothelial markers CD144/CD31 and functional LDL uptake assay of iECs on *day 21* using 5 different iPSC lines: CS0007iCTR-n07 (07n07), EDi028-A, C0002iCTR-n11 (02n11), EDi042-A, and CS0003iCTR-n14 (03n14). HUVECs and iPSCs are also included as controls. Scale bar = 150 µm. *C*: quantification of CD144/CD31 protein expression and LDL uptake dye (% positive cells of DAPI). Data are shown as means ± SE. Immunocytochemistry (ICC) and LDL uptake images shown here are representative results from 18 independent sites acquired, per iPSC cell line. EC, endothelial cell; ECGM, EC growth medium MV2; HUVECs, human umbilical vein endothelial cells; iECs, induced endothelial cells; iPSCs, induced pluripotent stem cells; VEGF, vascular endothelial growth factor.

Human iPSCs were induced to differentiate into nontissue-specific pan vascular endothelial cells (iECs). For this, pluripotent iPSCs were passaged as colonies using ReLeSR (STEMCELL Tech) and after 3 days, when they were small colonies of ∼60–200 µm size, cleaned out for any spontaneous differentiation (Supplemental Fig. S1*A*), the differentiation cocktail was initiated to induce iPSCs into primitive mesoderm (*phase I*) through the activation of the Wnt signaling pathway using CHIR99021, highly selective inhibitor of glycogen synthase kinase 3 (GSK-3), for 2 days (Fig. 1*A*). Afterward, the cells were induced into vascular progenitors (*phase II*) using the same media, supplemented with additional morphogens and growth factors including BMP4, FGF2, and VEGF_165_ for another 2 days (Fig. 1*A*). At *day 4* of differentiation, two different cell populations were observed: vascular progenitors at the periphery of the colonies and less differentiated pluripotent cells in the center of the colonies (Supplemental Fig. S1*A*). Cells were manually lifted (mostly the vascular progenitors at the periphery), singularized, and replated at a specified cell density (10,000–20,000 cells/cm^2^) with specific media to mature vascular progenitors into endothelial progenitors with supplementation of VEGF_165_ for 7 days (Fig. 1*A*). At *day 11* (Supplemental Fig. S1*A*), endothelial progenitors were again dissociated and replated at similar cell densities with the same media for another 10 days to enhance maturation and purification into iECs as shown in Fig. 1*A* (31).

This simple multistage differentiation protocol was repeated across five different iPSC lines to show robustness and reproducibility of the protocol. Figure 1*B* shows EC markers CD31^+^/CD144^+^ at *day 21* across all five cell lines, and Fig. 1*C* shows that a high percentage of cells expressed both markers (>80% of double expression in all cell lines). Additional EC markers were probed, and all lines presented VEGFA^+^/CD31^+^ and VEGFR2^+^/CD31^+^ protein expressions (Supplemental Fig. S1*B*). Importantly, we tested EC functionality in vitro at *day 21* through the functional assay Acil-Dil-LDL uptake (Fig. 1, *B* and *C*). LDL uptake by the iECs at this stage was routinely >40% (Fig. 1*C*). To further probe functionality, iECs were dissociated and replated onto a thick layer of Matrigel using low cell density, and after 24 h, iECs formed tube-like structures (Supplemental Fig. S1*C*), which were maintained after 48 and 72 h. These results show that we could establish an efficient and reproducible protocol to differentiate iPSCs into functional vascular iECs with high expression of the canonical vascular endothelial protein markers.

To understand the development of the iECs throughout the differentiation process, cells from multiple cell lines were collected in different days, and gene expression of main vascular EC markers was probed. Vascular mesoderm-specific transcription factor *ETV2* was measured (Supplemental Fig. S2*A*). iECs from all cell lines presented a higher expression of *PECAM-1* (CD31) and *VE-CADHERIN* (CD144) at *day 21* compared with that of *day 11*, and most of the cell lines presented higher expression of *KDR* (VEGFR2) at *day 21* compared with that of *day 11* (Supplemental Fig. S2, *B*–*D*). Importantly, iECs at *day 11* or *day 21* presented minimal or nondetectable mRNA expression of pluripotent markers Lin28 and *OCT4* compared with iPSCs at *day 11* or *day 21* (Supplemental Figs. S2*E* and S4*A*). These results show that the dissociating and extending the protocol from *day 11* to *day 21* further matured iECs. Gene expression of arterial (*NRP1* and *NOTCH1*) and venous genes (*EPHB4* and *NRP2*) was assessed in EC cultures. By *days 11* and *21*, both sets of genes were upregulated in the iEC cultures, suggesting neither a distinct arterial nor a venous phenotype of the iECs in vitro at these time points (Supplemental Fig. S3).

For a cGMP-compliant cell therapy to translate into the clinic, it is important that the final cell product be a defined homogeneous cell population. Although iECs generated here have >80% CD144^+^/CD31^+^-expressing cells by *day 21* (Fig. 1, *B* and *C*), to reach the greater purity with homogenous population of iECs expressing endothelial cell markers, we established the feasibility of magnetic-associated cell sorting (MACS) to enrich the final iECs population. *Day 21* iECs MACS-sorted for CD31 (Supplemental Fig. S4*C*) were probed for CD144 expression by flow cytometry and replated for immunostaining analysis at *day 23* (CD144/CD31 expression). Unsorted iECs presented around 92% of CD144^+^ expression at *day 21* (Supplemental Fig. S4*D*). Upon CD31-based MACS, this purity increased to 99.7% CD144^+^ iECs (Supplemental Fig. S4*D*), showing the feasibility of cell sorting to generate pure population of iECs for cell therapy applications. Sorted and unsorted iECs carried until *day 23* on differentiation continued to present high levels of CD144^+^ and CD31^+^ cells, as seen in Supplemental Fig. S4*E*.

### Proteomic Analysis Reveals iPSC-Derived Vascular ECs Exhibit a Similar Protein Profile as an Established Human Vascular EC Line (HUVEC Cells)

To gain knowledge from the iECs generated by this method and extensively define their protein expression profile, label-free quantitative proteomics using data-independent acquisition mass spectrometry was performed on *day 21* iECs generated from three different iPSC lines (03n14, EDi028-A, and EDi042-A). iPSCs (at *day 0*) from the same lines were used as negative control and HUVECs as a standard comparator. Before proteomics studies, we confirmed that HUVECs maintained an EC phenotype (Fig. 1, *B* and *C* and Supplemental Fig. S1, *B* and *C*). HUVECs presented high protein expression of main EC markers, such as VEGFA^+^/CD31^+^, VEGFR2^+^/CD31^+^ (Supplemental Fig. S1*B*), and CD144^+^/CD31^+^ (Fig. 1*C*), and they were also functional as probed by LDL uptake assay (Fig. 1*C*) and Matrigel-based tube formation assay (Supplemental Fig. S1*C*). These results made us confident to use this cell line as a valid primary endothelial cell comparator in the proteomics analysis.

**Figure 2.**
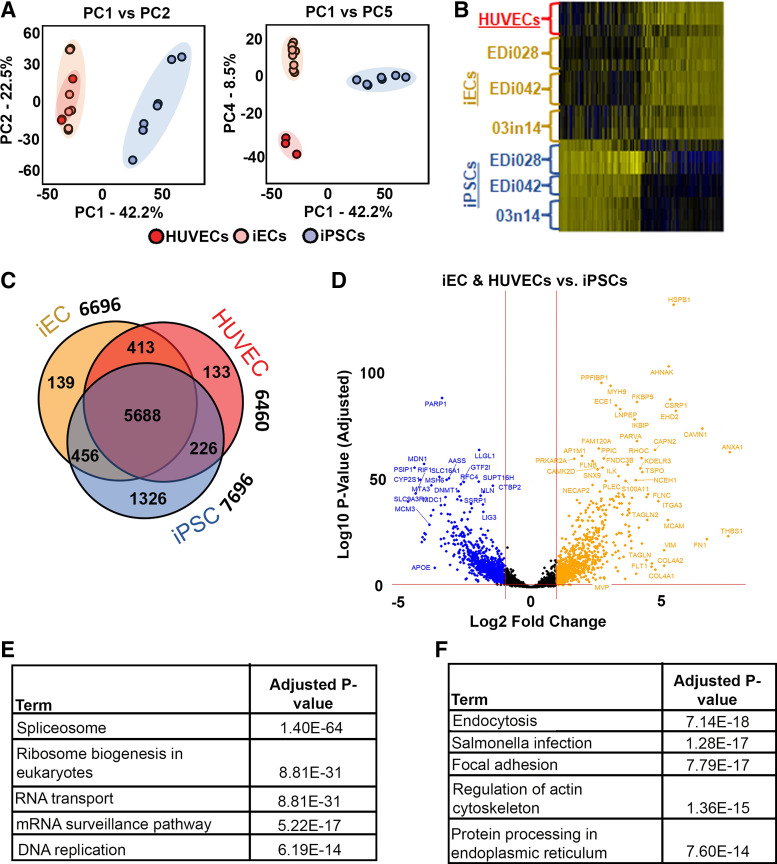
Extensive proteomic analysis reveals similarities between iECs and a human vascular EC line (HUVECs) protein expression. *A*: principal component analysis (PCA) of iECs *day 21* generated from 3 different iPSC lines (EDi028-A, EDi042-A, and 03n14), HUVECs, and iPSCs (EDi028-A, EDi042-A, and 03n14). *B*: differentially expressed proteins’ heatmap showing the relative expression levels of proteins differentially expressed with adjusted *P* values less than 0.01. *C*: Venn diagram showing the number of proteins whose expression is shared by all 3 types of cells, or between iECs and iPSCs only, iECs and HUVECs only, or iPSCs and HUVECs only. *D*: volcano plot of the log10-adjusted *P* value of each expressed protein on iECs and HUVECs vs. iPSCs. Transcripts that did not demonstrate differential expression with an adjusted *P* value of less than 0.05 and a log2 fold-change in either direction greater than 0.5 are plotted in black. Negative values (blue) are higher in endothelial cell types (iECs and HUVECs), whereas positive values (orange) are higher in iPSCs. *E*: enriched pathways seen in iPSCs only. *F*: enriched pathways seen in endothelial cell types (iECs and HUVECs). EC, endothelial cell; HUVECs, human umbilical vein endothelial cells; iECs, induced endothelial cells; iPSCs, induced pluripotent stem cells.

We identified a total of 20,852 proteins across all three types of cells, with 7,696, 6,696, and 6,460 proteins identified in iPSCs, iECs, and HUVECs, respectively. Principal component analysis (PCA) comparing iPSCs, iECs, and HUVECs demonstrates a distinct separation of two groups when running PC1 versus PC2. Importantly, iECs are grouped with HUVECs and they both were far from iPSCs (Fig. 2*A*), demonstrating that iECs presented a similar protein expression pattern to HUVECs and both of which are unique from the protein expression pattern of iPSCs. Not until PC5 did we achieve a distinct separation between iECs and HUVECs based on their proteomic profiles, implying that although they are endothelial cells, iPSC-derived ECs still present some unique protein expression that is distinct from HUVECs (Fig. 2*A*). The separation of protein expression pattern of iPSCs compared with that of iECs and HUVECs can also be observed on the heatmap generated (Fig. 2*B*), which supports the similarities between the protein expression pattern of HUVECs and iECs from three different cell lines. Interestingly, although a large proportion (*n* = 5,688) of proteins were detected within all the three cell types (Fig. 2*C*), 64.6% of the proteins showed statistically significant differential-expressed proteins (DEPs). Interestingly, iECs have roughly equivalent numbers of shared proteins with HUVECs (413, 6.2%) or with iPSCs (456, 6.8%), and HUVECs have a much smaller number of proteins uniquely shared with iPSCs (226, 3.5%). iPSCs contain a largely unique proteome (1,326, 17.2%). These data indicate that iECs are proteomically more similar to HUVECs than to iPSCs, demonstrating the success of the differentiation process.

Differential expression of proteins unique to endothelial cell type and iPSCs can be examined using the volcano plot in Fig. 2*D*, including the upregulation of proteins related to cellular bioenergetics (*APOE* and *PARP1*) in endothelial cell types (32, 33). In iPSCs, proteins related to cell survival (*HSPB1*) and intracellular regulations, such as plasma membrane organization (*EHD2*) and signal transduction (*MVP*), were upregulated. To gain insight into the functional implications of proteomic differences, we conducted a gene set enrichment analysis on the proteins upregulated in the iPSCs (Fig. 2*E*) as well as proteins upregulated in the endothelial cell types (Fig. 2*F*). The iPSC proteome was enriched with proteins involved in spliceosome and RNA transport, whereas endothelial cell types expressed proteins enriched for focal adhesion and actin cytoskeleton regulation. Full pathway enrichment results are provided in Supplemental Table S4. Overall, comparison of proteomic differences between iPSC and EC types demonstrated expected results, highlighting proteins and pathways consistent with a pluripotent state in iPSCs.

**Figure 3.**
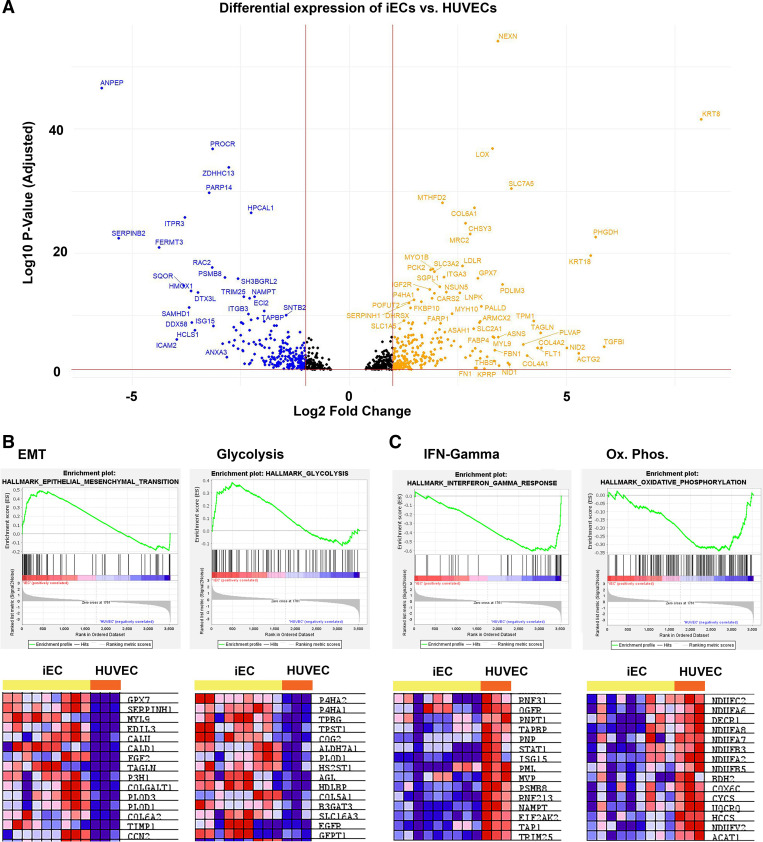
Differential expression of proteins in iECs vs. HUVECs demonstrated differences in functional pathways between the cell types. *A*: volcano plot of the log10-adjusted *P* value of each expressed protein on iECs vs. HUVECs. Negative values (blue) are higher in iECs, whereas positive values (orange) are higher in HUVECs. Epithelial-to-mesenchymal transition (EMT) and glycolysis pathways were upregulated in iECs (*B*), whereas IFN-γ and oxidative phosphorylation were upregulated in HUVECs (*C*). ECs, endothelial cells; HUVECs, human umbilical vein endothelial cells; iECs, induced endothelial cells.

Perhaps of greater interest is the comparison of proteins that differentiate what may be considered “mature” ECs (HUVECs) from the stem cell-derived iECs, as these differences may reveal targets for further maturation or key functional differences between these two cell types. This comparison found 4,634 DEPs (Fig. 3*A*), with comparative pathway analysis on DEPs revealing multiple differential functional pathways between the cell types (Supplemental Table S5), including prominent upregulation of epithelial-to-mesenchymal transition (EMT) and glycolysis in iECs (Fig. 3*B*) and concomitant upregulation of IFN-γ (along with other inflammatory pathways) and oxidative phosphorylation in HUVECs (Fig. 3*C*). Proteins that were most upregulated in iECs included proteins related to nucleic acid metabolism (*SAMHD1* and *DDX58*), TNF-α signaling via NF-κβ (*DDX58* and *SERPINB2*), and IL-6 and IL-10 signaling (*HMOX1*). In HUVECs, proteins related to focal adhesions (*FLT1* and *COL4A2*) and VEGF signaling (*FLT1*) were highly upregulated (Supplemental Tables S6 and S7). Thus, gene set enrichment analysis identified pathways of interest in both iPSCs and endothelial cell types and demonstrated that iECs and primary HUVECs exhibit proteomic similarities important in maintaining endothelial cell function. Notably, the endothelial cell types contain similarities in key proteins and enrichment in pathways not present in iPSCs.

### Screening of Abundant Posttranslational Modifications Reveals Differentially Modified Peptides across Differentiation States

**Figure 4.**
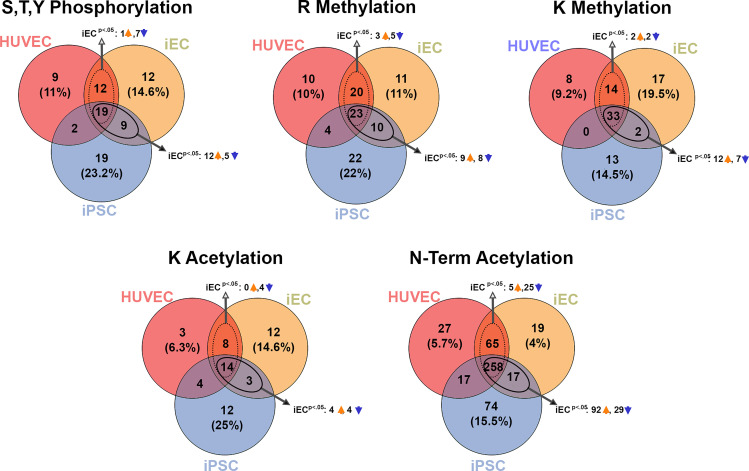
Posttranslational modification (PTM) analysis reveals more shared PTMs between iECs and HUVECs and fewer between endothelial cell types and iPSCs. Phosphorylation, R and K methylation, K and *N*-term acetylation sites in HUVECs, iECs, and iPSCs were compared. Note: the methylated CTNNB peptide was only observed in 2 of 8 total iPSC replicates. DEP, differential-expressed protein; HUVECs, human umbilical vein endothelial cells; iECs, induced endothelial cells; iPSCs, induced pluripotent stem cells.

Total proteome can provide detailed insight into the overall cell phenotype, but additional granularity into cell signaling and regulatory states may be gleaned from specific interrogation of PTMs between cells. Although PTMs are typically difficult to detect without prior enrichment due to stoichiometric limitations, we were able to confidently identify several PTM sites across the cell lines (Fig. 4 and Supplemental Table S11). Of note, PTM sites tended to be more similar between iECs and HUVECs, with fewer shared PTMs between the endothelial sites and iPSCs (Fig. 4). To glean insight into the functional implications of differential PTM findings, gene ontology analysis on proteins found in each PTM category was conducted with DAVID gene ontology platform. For most PTMs, modified proteins related to cell adhesion and focal adhesion pathways were prevalent across the EC cell types, whereas PTMs on proteins related to chromatin and RNA binding were more dominant in iPSCs (Supplemental Figs. S5, S6, S7, S8, and S9).

**Figure 5.**
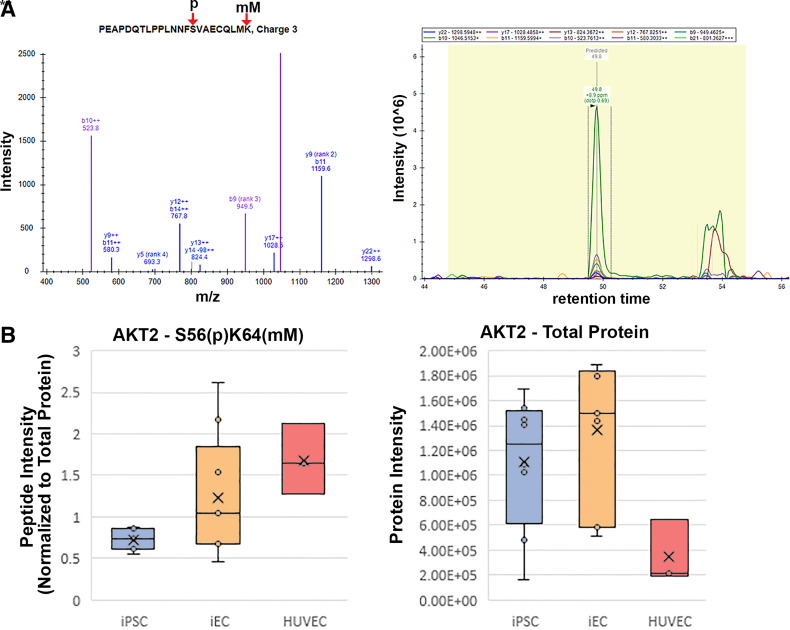
Dual phosphorylation and lysine monomethylation site on Akt2 was elevated in HUVECs as compared with iECs. *A*: intensity peaks demonstrate phosphorylation and lysine monomethylation on Akt2. *B*: levels of the modified Akt2 site were elevated in HUVECs as compared with iECs while iECs contained the largest total amount of Akt2. Significance of DEP examined using two-tailed *t* tests, **P < 0.001; ns, nonsignificant. DEP, differential-expressed protein; HUVECs, human umbilical vein endothelial cells; iECs, induced endothelial cells; iPSCs, induced pluripotent stem cells.

**Figure 6.**
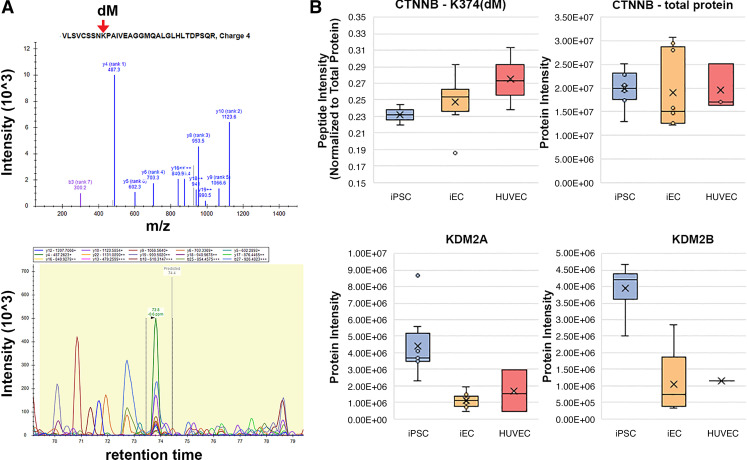
Lysine monomethylation site on *CTNNB1* was only present in endothelial cell types (iECs and HUVECs). *A*: intensity peaks demonstrate lysine monomethylation of *CTNNB1*. *B*: lysine methylation of *CTNNB1* was present primarily in endothelial cell types (iECs and HUVECS), whereas KDM2A and KDM2B were present in higher levels in iPSCs. HUVECs, human umbilical vein endothelial cells; iECs, induced endothelial cells; iPSCs, induced pluripotent stem cells.

As is true for most PTM profiling studies, inferring functional implications for observed PTMs is a significant challenge, given limitations in databases that annotate functional impact of a large number of PTM sites. That being said, the granularity that PTM profiling can provide into cellular signaling state provides the opportunity to identify potential targets for further method development toward terminal iEC differentiation in vitro. For instance, we profiled the phosphorylation sites that demonstrated significant differences between the different cell types using the piNET platform (34) to identify upstream kinases known to regulate observed phosphorylation sites. Although many sites currently have no annotated regulatory kinase, we did observe several sites regulated by cyclin-dependent kinase 1 (CDK1), whose phosphorylation was increased in iPSCs (Supplemental Fig. S10*A*). This observation serves as a sanity check for these data, as CDK1 has a well-known role in maintaining pluripotency (35). A handful of other sites also had known regulatory kinases, driving abundance changes between iECs and iPSCs, including two upregulated sites linked to homeodomain-interacting protein kinase (HIPK2), a kinase that has previously been studied for a role in modulating endothelial cell differentiation and angiogenesis (36). Only three differential sites between iECs and HUVECs are linked to known regulatory kinases (Supplemental Fig. S10*B*), implicating members of the PKC and CDK family of kinases in their differential phosphorylation. In addition to these general trends, a handful of particularly notable PTMs were observed. We observed a dual phosphorylation (S56) and lysine monomethylation (K64) site on the protein Akt2 that was significantly elevated in HUVECs versus iECs, despite the iECs expressing the largest total amount of Akt2 protein (Fig. 5 and Supplemental Figs. S5 and S7). Another potentially impactful PTM difference was observed on β-catenin (*CTNNB1*) (an effector of WNT that is present in transcription factor binding/signaling pathways), with lysine methylation at 354, which is only present in the endothelial cell types. Interestingly, the demethylase enzymes KDM2A and KDM2B that are known to act on *CTNNB1* methylation at this site showed a reciprocal expression pattern (Fig. 6 and Supplemental Fig. S7). Potential functional implications of these differential PTM sites are examined in the Discussion. Overall, the PTM screening study corroborates our conclusions from total proteome analysis that the iECs are distinct from iPSCs in PTM profile, with more shared abundant PTMs with HUVECs than with their parental iPSCs.

## DISCUSSION

In the present study, we report a simple and reliable method to generate ECs from iPSCs in adherent cultures in a defined differentiation media using small molecules and growth factors as compared with previously published methods. This method is very efficient and consistent across multiple iPSC lines derived from different donors giving rise to functional iECs with high efficiency within 3 wk from iPSC stage. To arrive at this robust iEC differentiation method, various critical process parameter optimizations were performed to initiate mesodermal induction that included optimization of starting iPSC colony size, seeding cell densities, culture media, timing of differentiation, cell harvesting and replating, and cell sorting. For example, initiating iEC differentiation with small iPSC colonies with chemical methods resulted in a consistent process with more reliable and efficient iEC differentiation compared with when colonies were larger than 200 µm (data not shown). Another important aspect of this study is the development of a protocol that is favorable for regenerative cell therapy applications, by producing a terminally differentiated iEC desirable target cell population devoid of nontarget highly proliferative cells in the final cell therapy drug product.

Specifically, to differentiate iPSCs into iECs, activation of the canonical Wnt/β-catenin signaling pathway is a strategy widely used to give rise to mesodermal cells from embryonic state-like cells (37, 38), and vascular endothelial lineages are derived from mesoderm (31). In this method, we activated the canonical Wnt signaling pathway through the inhibition of GSK3-mediated β-catenin phosphorylation (39) using the small molecule CHIR99021. It is known that a short pulse-active Wnt/β-catenin signaling from iPSC stage enhances expression of mesodermal genes by supporting an exit from pluripotency (40). On the other hand, inhibiting the Wnt pathway leads to the failure to generate fetal liver kinase 1 (*Flk1^+^*, or *VEGFR2*) mesodermal precursors and subsequent mature mesodermal lineages (37). Once mesodermal specification is performed from pluripotency, use of basic FGF (FGF2) induces specification of the hemangioblast precursor of ECs, and the use of BMP4 is known to induce expression of *Flk1/VEGFR2* (31). At this stage, stem cell leukemia (SCL) and the adherens junction protein vascular endothelial (VE)-cadherin, markers of endothelial precursors, are also induced. *Flk1*/*SCL^+^* hemangioblasts within the mesoderm form blood island clusters and are induced by vascular endothelial growth factor (VEGF)-A (31), a critical morphogen known to drive specification of vascular endothelium (41). In this study, we used these critical morphogens, namely FGF2, BMP4, and VEGF-A, to induce vascular endothelial cell progenitors from mesodermal cells. During this vascular mesoderm specification stage, a heterogenous population of cells are formed in the blood islands and undergo into different directions: the outer cells flatten and become ECs, whereas the inner cells differentiate into hematopoietic cells (31). We also observed two distinct populations at this stage of differentiation—peripheral cells at the edge of an iPSC colony that have a cobblestone-like morphology and cells in the center of the colonies that have a distinct morphology. The cobblestone-like cells lift easily from the plate and are replated to continue the vascular endothelium differentiation onto the next stages, when the endothelial lineage cells continue to be matured with the addition of VEGF-A and other media growth supplements. At *day 11*, iECs are dissociated and replated and further matured with the continued use of VEGF-A until final cell harvest. During human development, the ECs that will comprise the arteries are close to the notochord, a source of Sonic hedgehog that induces high levels of VEGF. On the other hand, to achieve a venous fate, lower Sonic hedgehog levels act to induce low levels of VEGF (31). Our method gave rise to a mixed EC population, displaying arterial and venous lineage markers similar to other studies (42). Future studies may manipulate Sonic hedgehog pathway and concentrations of VEGF to direct the iECs into arterial or venous fate (43). The method reported here routinely results in a high percent of the iEC population (>80%) expressing CD31 and CD144 by *day 21* across multiple iPSC lines, which can be enriched >99% purity for iEC markers using MACS for CD31 cell surface protein. An early report also explored the differentiation of multiple iPSC lines into iECs and showed that iPSCs derived from skin fibroblasts and differentiated into iECs in 10 days presented a population expressing VEGFR2^+^, CD31^+^, and CD144^+^ in 1%–5% of the cells; after sorting, iECs expressed more EC markers, formed network-like structures, and had a cobblestone morphology (44). Since protein expression is highly informative about cell state, phenotype, and function of ECs and given the fact that there are currently no studies that have examined how the proteomic state of iPSC-derived ECs, we conducted an in-depth proteomic analysis using iECs derived from multiple donor iPSC lines. We believe that this information is critical for further development of iPSC-derived endothelial cells as they are being translated from the bench toward clinical regenerative cell therapy applications.

The proteomic comparison of iECs, iPSCs, and HUVECs represents, to our knowledge, the most comprehensive if not the only of its kind to date. Principal component analysis demonstrated clearly that the proteomic state of iECs was more similar to HUVECs than iPSCs, supporting our conclusions made from analysis of individual protein markers and RNA expression that this iPSC differentiation method was effective and efficient. This is quite significant, given the known variability associated with iPSC-derived cell models that can be confounded by donor variability and genetic stability of iPSCs among various factors (45). Functional pathway analysis revealed several intriguing observations. For instance, proteins associated with spliceosomes were upregulated in iPSCs, agreeing with previous findings (46). As a splicing factor switch occurs during the differentiation of mesodermal cells to endothelial progenitor cells (47), further investigation may lead to an increased understanding of differentiation processes and mechanisms to control cell fate. In endothelial cell types, focal adhesions and regulation of actin cytoskeleton were upregulated. Focal adhesions play an important role in endothelial cell morphology and function, anchoring cells to the vascular wall and aiding in vascular responses to physical and chemical stimuli (48, 49). Taken together, we concluded that the proteomic differences between iPSCs and ECs were consistent with their highly disparate functional states. We then turned to the functional pathways that differentiate proteomic states of iECs from HUVECs, as these pathways may provide targets for further maturation of iECs. Interestingly, proteomic data implied that although mitochondrial metabolism/oxidative phosphorylation was upregulated in HUVECs, the iECs expressed proteins more consistent with a dominant glycolytic state. Together these data indicate that there may be key differences in metabolic function between nascent iECs and primary tissue-derived ECs and that interventions to promote mitochondrial function and oxidative metabolism may assist in further development of iECs. Total protein data implied the upregulation of proinflammatory interferon signaling in HUVECs relative to more naïve iECs. The strong interferon signaling signature that was enriched in HUVECs may reflect their prior development within an intact human system, where ECs participate in immune surveillance and response (50). In the future, it will be of interest to explore whether exposure to immune-related inflammatory signals is a key component of EC maturation or an auxiliary finding.

Posttranslational modification is a major mode for regulating protein-protein interactions and enzyme activity (51). We were able to detect hundreds of PTMs across our proteins without a priori chemical enrichment. From our survey, PTMs on proteins related to many pathways intrinsic to iPSC and EC function were identified with a handful demonstrating interesting abundance patterns that may implicate regulation of important EC differentiation pathways. Among these, we highlighted and confirmed from the raw data a dual methylation and phosphorylation on Akt2 (phosphorylated serine 56 and monomethylated lysine 64) as well as dimethylation of lysine 374 on β-catenin. Akt2 is a major regulator of metabolic pathways, whereas β-catenin is a critical mediator of Wnt signaling. Since metabolic-related proteomic differences were prominent between HUVECs and iECs and, as discussed earlier, Wnt signaling is critical for the efficient differentiation of iECs from iPSCs, these two PTM sites were highlighted for in-depth consideration. Little is known regarding the functional implications of the PTM sites quantified on Akt2. Neither of these residues have existing annotation for these PTMs, though the methylation site may impact a known ubiquitinylation site and thus impact protein stability. Akt2 is involved in metabolic regulation (52, 53) and cell cycle progression (54), thus this differential PTM could reflect altered Akt2 activity pertinent to functional iEC and HUVEC differences. Future studies manipulating these specific residues on Akt2 in iECs and HUVECs will test whether these dual PTMs are functionally relevant to EC differentiation and maturation.

The dimethylation site identified on β-catenin is supported, albeit minimally, by functional data in the literature. Specifically, degradation of nuclear β-catenin depends on *KDM2A*-induced demethylation, which presumably promotes reduced transcriptional activity of this end effector of the Wnt signaling pathway (55). Our observation of increased dimethylation of K374 coupled with reduced expression of *KDM2A* and *B* in endothelial cell types indicates that enhanced methylation may promote nuclear stabilization of β-catenin and promote efficient differentiation of iECs. This information can be used to further explore the role of Wnt signaling in iEC differentiation and maintenance of EC function and highlights either the demethylases *KDM2A* and *B* or the as-yet-unknown lysine methyl transferase acting on β-catenin as potential molecular targets to improve iEC differentiation efficiency in future studies.

In summary, we report a robust, improved, and highly efficient method for the differentiation of iECs across multiple donor human iPSC lines, which provides excellent promise as a cellular therapeutic development for vascular applications. We have extensively characterized both the total and abundant PTM proteomes of the iECs produced and shown that although the iEC are highly similar to a primary reference ECs (HUVECs), there are subtle proteomic differences that provide targets for further study and enhancement with the goal of achieving functionally potent iECs for ultimate therapeutic benefit in cell therapy applications.

## ETHICAL APPROVALS

Human cell lines were obtained or created at Cedars-Sinai under the auspices of the Cedars-Sinai Medical Center Institutional Review Board (IRB)-approved protocols. Specifically, the iPSC cell lines and differentiation protocols in the present study were carried out in accordance with the guidelines approved by Stem Cell Research Oversight (SCRO) committee and IRB, under the auspices of IRB-SCRO Protocols Pro00032834 (iPSC Core Repository and Stem Cell Program) and Pro00036896 (Sareen Stem Cell Program). In vitro studies using human cell lines were conducted from participants who provided written informed consent for research studies. Remaining studies were conducted with postmortem human specimens with appropriate IRB approvals.

## DATA AVAILABILITY

Proteomic data (raw and processed) are openly available on the ProteomeXChange repository (https://www.proteomexchange.org; identifier PXD038493).

## SUPPLEMENTAL DATA

10.6084/m9.figshare.21668129 Supplemental Figs. S1–S10 and Supplemental Tables S1–S11: https://doi.org/10.6084/m9.figshare.21668129.

## GRANTS

This work was supported by Cedars-Sinai Programmatic Funds (to D.S.). Part of this effort was funded under Medical Technology Enterprise Consortium (MTEC) solicitation MTEC 20-07-QualRegen-010 with funding from US Army Medical Research and Development Command. N.R.A.’s salary is funded by a California Institute for Regenerative Medicine (CIRM) postdoctoral fellowship.

## DISCLAIMERS

The funders were not involved in the study design, collection, analysis, interpretation of data, the writing of this article, or the decision to submit it for publication. The data and views expressed in this article are those of the authors and may not reflect the official policy or position of the Department of the Army, Department of Defense, or the US Government.

## DISCLOSURES

No conflicts of interest, financial or otherwise, are declared by the authors.

## AUTHOR CONTRIBUTIONS

N.R.A., R.d.S.S., and D.S. conceived and designed research; N.R.A., R.d.S.S., A.G., A.A.-S., S.K., and S.E. performed experiments; N.R.A., R.d.S.S., A.G., and S.K. analyzed data; N.R.A., R.d.S.S., and A.A.-S. interpreted results of experiments; N.R.A., R.d.S.S., and S.J.P. prepared figures; R.d.S.S. drafted manuscript; N.R.A., S.J.P., and D.S. edited and revised manuscript; S.J.P. and D.S. approved final version of manuscript.
